# The iatrogenic injury of double vena cava due to misdiagnosis during the radical nephroureterectomy and cystectomy

**DOI:** 10.1186/s12957-015-0469-x

**Published:** 2015-02-12

**Authors:** Ye-Qing Mao, Shao-Xing Zhu, Wei Zhang

**Affiliations:** Department of Urology, The First Affiliated Hospital of College of Medicine, Zhejiang University, Zhejiang, People’s Republic of China; Department of Urology, Zhejiang Cancer Hospital, 38 Guangji Road, Hangzhou, 31002 Zhejiang People’s Republic of China

**Keywords:** Double inferior vena cava, Vascular reconstruction, Iatrogenic injury, Misdiagnosis, Anastomosis

## Abstract

Double inferior vena cava (d-IVC) is a subtype of vascular anomaly that rarely needs treatment. Here, we present a rare case of d-IVC accompanied with concurrent renal pelvis and bladder carcinoma. Due to misdiagnosis, the anomalous left inferior vena cava (IVC) entering the left renal vein was mistaken as the gonadal vein and was then severed during the radical nephroureterectomy. Fortunately, the injured left IVC was recognized correctly during the following cystectomy. The vascular reconstruction operation was performed to recanalize the left iliac veins by anastomosing the ligated vascular stump to the right IVC in an ‘end-to-side’ way. During the hospitalization, the patient was treated with ‘low molecular weight heparin’ and then warfarin to ensure an ideal international normalized ratio. He recovered well from the surgery. A meticulous and comprehensive analysis of radiographic imaging is critical to avoid misdiagnosis of d-IVC.

## Background

The inferior vena cava (IVC) stems from three pairs of embryonic veins (posterior cardinal, subcardinal, and supracardinal veins), whose embryogenesis involves development, regression, anastomosis, and replacement of them [1]. Once any step among the processes is hindered, the development of IVC will inevitably suffer, leading to a series of vascular anomalies.

Double-IVC (d-IVC), one of the most well-described anomalies of IVC, has been reportedly detected in approximately 0.2 ~ 3% of the population [2,3]. Its pathogenesis is generally attributed to the failure of regression of the left supracardinal vein. Although d-IVC has been reported to accompany with at least four types of pelvic venous variation, the duplicated left IVC generally drains into the left renal vein and then enters normally into the right IVC without other incidental anatomic variations just like our case [4].

Most cases of anomalous IVC including d-IVC are recognized pre- or intraoperatively and normally left untreated. Here, we present a case of inadvertent injury of d-IVC due to carelessness by the radiologists and surgeons, which nearly caused severe surgical complications.

## Case presentation

**Figure 1:**
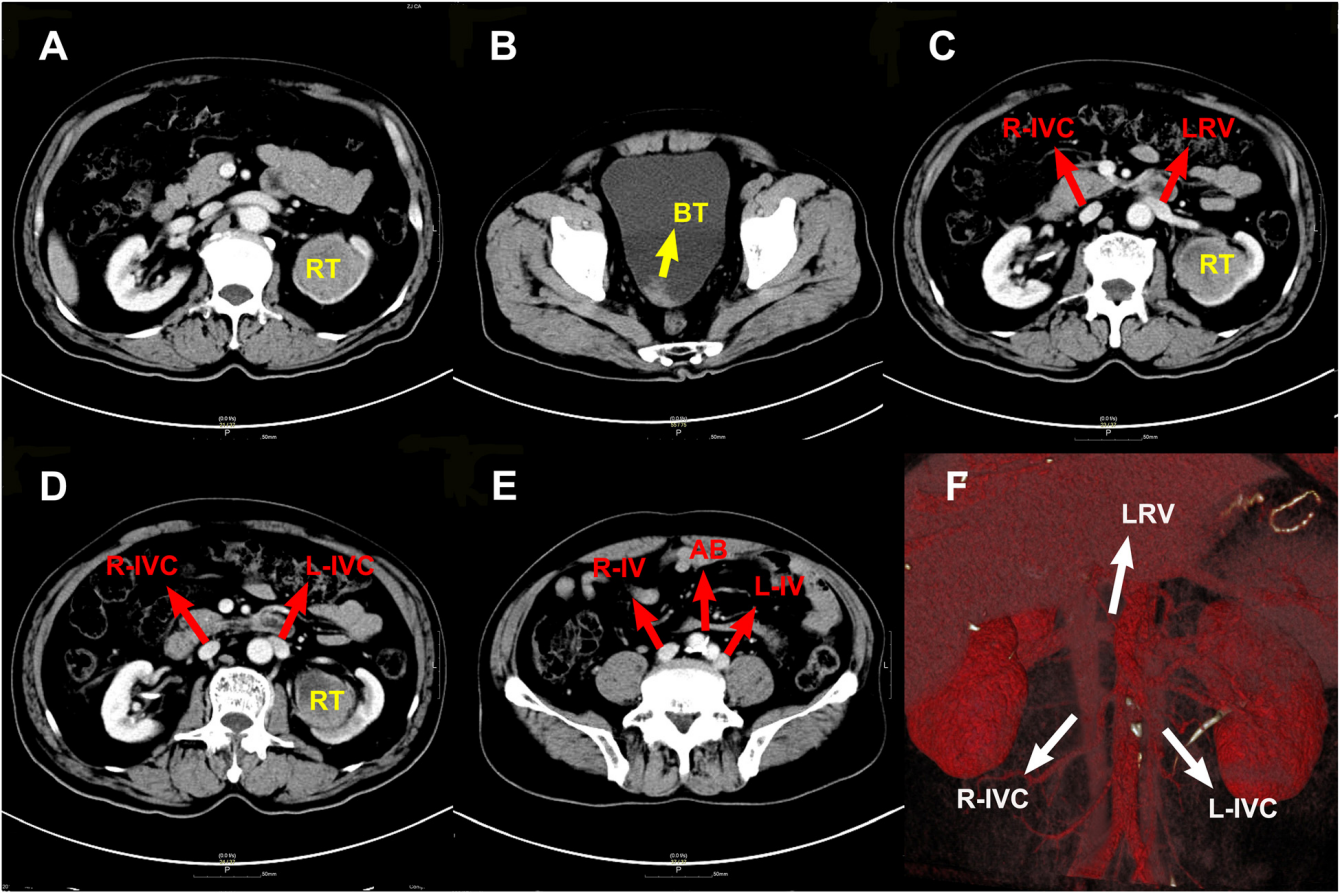
**The computerized tomography images and three-dimensional reconstructed image. (A)** The renal tumor (RT). **(B)** The bladder tumor (RT). **(C)** The left renal vein (LRV) entering the right inferior vena cava (R-IVC). **(D)** The right and left inferior venae cavae (L-IVC) are visible. **(E)** The supposed confluent point of common iliac veins is not found near the aortic bifurcation (AB). **(F)** The three-dimensional reconstructed image.

A 63-year-old man was referred to our department for complaint of intermittent gross hematuria. His vital signs were normal; the red blood cell urine test was marked ‘++++’ and exfoliated tumor cells were found in urine, with other laboratory tests unremarkable. The computed tomography (CT) and ultrasonography (US) scan detected an occupying lesion within the left kidney (Figure 1A) and a neoplasm measuring 5.0 × 7.0 cm at fundus of the bladder (Figure 1B) that was confirmed by cystoscopy later. Both lesions were pathologically diagnosed with urothelial carcinoma (high grade) through biopsy. No visible enlarged lymph nodes were identified. Therefore, a radical nephroureterectomy combined with radical cystectomy was selected to perform.

Under general anesthesia, the patient was placed in flank position. A 20-cm long 11th intercostal incision was made to better visualize the left kidney and ureter. When freeing the left renal pedicle, we found a large branch entering the left renal vein cephalad along with the aorta laterally. The vessel, measuring 1.3 cm in width, was mistaken as a dilated gonadal vein and therefore ligated.

After removing the affected kidney and ureter, the patient was repositioned in supine position for radical cystectomy. The surgery was performed through a hypogastric midline incision. When the left sidewall of bladder was retracted and the left iliac vessels were scrutinized for lymphadenopathy, we noticed the remarkably distended external iliac vein. Further dissecting the cephalad, the iliac vein was found in solitary route rather than confluence with the contralateral counterpart at the aortic bifurcation. Retrospectively, as the preoperative CT images displayed, the mis-ligated branch of the left renal vein went down to become the ipsilateral iliac vein without visible collateral vasculature with the contralateral veins, which indicated a case of d-IVC anomaly (Figure 1C,D,E). These images were later submitted to radiologists for three-dimensional reconstruction (Figure 1F).

**Figure 2:**
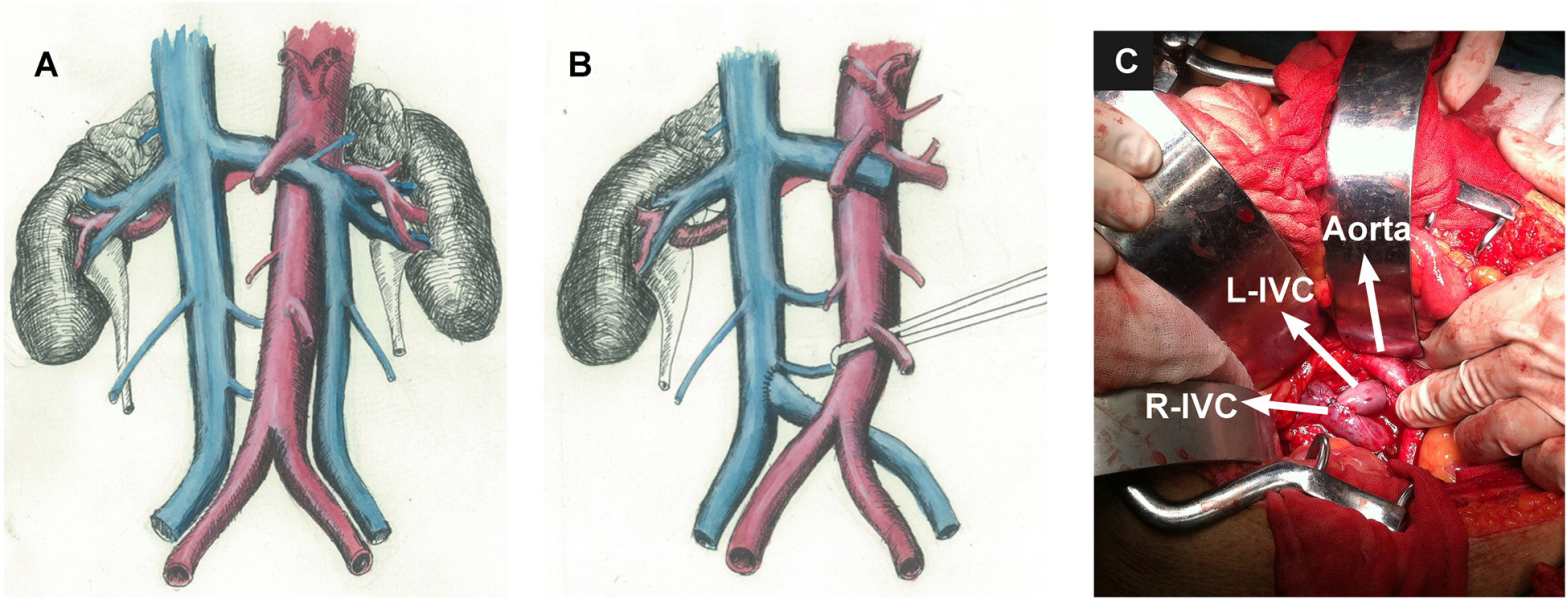
**The schematic rendering and photograph of the anomaly and reconstruction. (A)** The left inferior vena cava (L-IVC) entering the left renal vein anomaly. **(B)** The accidentally injured left IVC was anastomosed with the right IVC (R-IVC) to restore the ipsilateral iliac vasculature. **(C)** The photograph of the operation.

After the bladder was removed, the intended urinary diversion procedure was suspended for preferential treatment of vascular reconstruction. Prior to the treatment, the patient was intravenously injected with heparin (1.0 mg/kg) to prevent presumed thrombosis formation. Then, the aortic bifurcation was dissected and freed, to make up sizable posterior space. The ligated vessel stump or the anomalous left IVC was then pulled to the right side beneath the bifurcation and anastomosed in an ‘end-to-side’ way to the right IVC by 5/0 Prolene suture (Figure 2). Then, we went on to resume the urinary operation, which ended up with the ileal replacement of bladder (Bricker operation) as the urinary diversion method.

Postoperatively, the patient was treated with ‘low molecular weight heparin’ at dosage of 4,000 IU by subcutaneous injection twice daily for some days until recovery of gastrointestinal function; then, the ‘low molecular weight heparin’ was changed to warfarin to ensure the international normalized ratio (INR) at 2 ~ 2.5 s. The patient underwent an uneventful course without complaint of extremity pain or swelling and was discharged 14 days later.

Like most vascular variations, a d-IVC *per se* rarely causes symptoms, so most of the cases are often incidentally diagnosed by imaging for other reasons or by chance during operations. It has been well acknowledged that the presence of d-IVC might complicate surgery resulting in intractable bleeding. Reports of iatrogenically injured anomalous IVC are sparse. Shindo *et al*. reported four cases of aortic aneurysm with concomitant IVC anomalies; during the aortic surgery, one of them suffered from great blood loss due to laceration of the aberrant left-sided IVC [5].

The case we present here describes an iatrogenic injury of d-IVC resulted from insufficient preoperative radiological assessment. Generally, diagnosis of d-IVC is not too difficult by means of CT scanning or magnetic resonance imaging, but there have been several reports of malpractice caused by misjudgment or negligence. Some patients were reported to undergo unnecessary exploration to find presumed metastatic testicular carcinoma, due to the similar appearance of d-IVC to lymphadenopathy on cross-images [6,7]. In our case, the left-side IVC was misidentified as a gonadal vein in that the relationship between the ‘vessel’ and the ipsilateral iliac vein was ignored. Thanks to the successive pelvic operation providing good exposure of the anomalous vessel, we took measures to remedy the accidental error in time.

There is no standard procedure for treatment of such iatrogenic injured IVC. Shindo *et al*. treated the ruptured d-IVC by simple suture ligation [5]. The reason why they did not choose vascular reconstruction is not clear; maybe it was the entrapment of d-IVC within the inflammatory aneurysm that made the vessel stump unfit for repairment or anastomosis. Here, we anastomosed the severed left IVC to the right IVC beneath the aortic bifurcation to restore the normal vasculature. In addition, for fear of potential deep venous thrombosis resulting from iliac vein lesion caused by compression of right common iliac artery against lumbar spine, we lay emphasis on anticoagulation measures [8]. Dose adjustment of warfarin to maintain an ideal INR and regular US scanning on IVC bifurcation are critically important. In addition, frequent observation of complexion, skin temperature, and pain of affected extremity is absolutely necessary.

## Conclusions

Despite a group of asymptomatic congenital diseases that rarely need treatment, anomaly of IVC holds clinical implication to surgeons; inadvertent injury of these aberrant veins may complicate surgery unexpectedly. Therefore, a meticulous and comprehensive analysis of radiographic imaging is the preventative measure to avoid misdiagnosis.

## Consent

Written informed consent was obtained from the patient for publication of this case report. A copy of the written consent is available for review by the Editor-in-Chief of this journal.

## Abbreviations

d-IVC: double inferior vena cava
CT: computed tomography
US: ultrasonography
INR: international normalized ratio
